# The Effects of Acupressure on Improving Health and Reducing Cost for Patients Undergoing Thoracoscopic Surgery

**DOI:** 10.3390/ijerph19031869

**Published:** 2022-02-07

**Authors:** Hsing-Chi Hsu, Kai-Yu Tseng, Hsin-Yuan Fang, Tzu-Min Huang, Chi-Chung Kuo, Li-Li Chen, Wei-Fen Ma

**Affiliations:** 1 Department of Nursing, Hungkuang University, Taichung 433304, Taiwan; chiqueens@sunrise.hk.edu.tw; 2 Department of Nursing, Central Taiwan University of Science and Technology, Taichung 406053, Taiwan; 107179@ctust.edu.tw; 3 School of Medicine, China Medical University, Taichung 406040, Taiwan; fanghy@mail.cmuh.org.tw; 4 Department of Surgery, China Medical University Hospital, Taichung 406040, Taiwan; D22690@mail.cmuh.org.tw; 5 Department of Neurology, Taichung Tzu Chi Hospital, Buddhist Tzu Chi Medical Foundation, Taichung 427213, Taiwan; tc1772601@tzuchi.com.tw; 6 School of Post-Baccalaureate Chinese Medicine, Tzu Chi University, Hualien 970302, Taiwan; 7 School of Nursing, College of Health Care, China Medical University, Taichung 406040, Taiwan; 8 Department of Nursing, China Medical University Hospital, Taichung 406040, Taiwan; 9 PhD Program for Health Science and Industry, China Medical University, Taichung 406040, Taiwan

**Keywords:** acupressure, anxiety, comfort, cost-effective, physical health, thoracoscopic surgery

## Abstract

Objectives. This study aimed to assess the effectiveness of practicing acupressure on the Shenmen and Neiguan acupoints with a view to reduce anxiety and improve the comfort and physical health of patients undergoing thoracoscopic surgery. Methods. A total of 100 hospitalized patients undergoing thoracoscopic surgery were assigned randomly into the experimental (*n* = 49) and control groups (*n* = 51). Subjects in the experimental group received routine care plus acupressure on the Shenmen and Neiguan acupoints, while those in the control group received regular routine care. The data were collected using demographic information, physical and surgical data, the Visual Analog Scale (VAS)-A, the State-Trait Anxiety Inventory Y Form (STAI-Y1), and Shortened General Comfort Questionnaire scores. The linear mixed model was used to examine the influences of acupressure on VAS-A and STAI-Y1 scores at different time points before and after the surgery to observe group-by-time interactions. Results. The mean age of the subjects was 60.97 years. All subjects had mild-to-moderate anxiety after surgery and showed a statistically significant decline in regression coefficients on the first and second days after the intervention (β = −11.61, *p* = 0.002; β = −18.71, *p* < 0.001). Similarly, for STAI-YI scores, the data showed a significant difference in the pre-test and post-test interactions between the two groups (β = 4.72, *p* = 0.031). Conversely, acupressure did not have a statistically significant difference on comfort (F = 2.953, *p* = 0.057). Compared with the control subjects, the experimental subjects used less morphine and developed side effects less frequently (*p* < 0.01). They were also able to get out of bed after surgery 163.79 min earlier (*p* < 0.05). Conclusions. Acupressure is a simple and easy-to-practice treatment. Acupressure on the Shenmen and Neiguan acupoints reduces anxiety and improves recovery in patients after undergoing thoracoscopic surgery.

## 1. Introduction

Anxiety is a feeling of apprehension and uneasiness triggered by uncertainty about a possible threat or worries about unknowns in the future [1,2]. More than 60% of patients experience anxiety postoperatively [3]. Moreover, pain, indwelling tube placement, reduced mobility, and other factors may aggravate patients’ emotional distress and cause anxiety and fear [4]. These negative emotions can even drastically impact postoperative surgery and prolong hospitalization [5]. Therefore, in addition to pain control, effective postoperative anxiety management is important in improving patients’ quality of life [6].

Recent studies have proven the effectiveness of nonpharmaceutical intervention and complementary medicine in mitigating anxiety in patients undergoing thoracoscopic surgery. For example, music therapy has been shown to help stabilize heart rhythm and reduce anxiety [7]. However, music therapy has its limits as it is not particularly cost-effective considering the environment, equipment, and highly professional skills needed for its effective practice [8]. Based on the theory of meridians in traditional Chinese medicine, acupressure, often called “acupuncture without needles,” requires the use of a practitioner’s fingers to apply pressure to the acupoints across the meridians to reactivate blocked energy and facilitate the flow of Qi in the patient’s body [9,10]. It is safe, easy to practice, and cost-reducing, and has been extensively used as a complementary or alternative intervention for pain control in patients undergoing thoracoscopic surgery.

Acupoint activation energizes myelinated nerve fibers to stimulate the hypothalamus and pituitary gland, resulting in the release of endogenous opioids. It also helps to increase the secretion of serotonin and norepinephrine and to lower the serotonin and adrenocorticotropic hormone levels, thereby reducing anxiety by regulating neurotransmitter concentrations [11]. With meridians connecting every organ, applying pressure to specific acupoints along the meridians reduces preoperative anxiety [12]. Acupoint activation has also been found to be empirically capable of increasing sympathetic nerve activity [12,13] and alleviating anxiety in adult patients in different treatment settings [14]. In their study on the efficacy of acupressure on Shenmen and other acupoints in reducing the anxiety and physical discomfort of patients with cancer undergoing bone marrow biopsy and aspiration, Rizi et al. found that the anxiety level of the experimental subjects was lower than that of the control subjects (1.5 ± 0.5, *p* = 0.018) [15]. The effectiveness of acupressure in promoting sympathetic nerve activity, regulating heart rate, and lowering blood pressure has also been empirically proven by another study [13].

Despite the empirically supported effectiveness of acupressure in improving mental and physical health, studies on the application of acupressure for pain management in patients undergoing thoracoscopic surgery are lacking. To this end, our experimental study aimed to examine the effectiveness of acupressure in reducing the anxiety and improving the comfort and physical wellbeing of patients undergoing thoracoscopic surgery.

## 2. Materials and Methods

### 2.1. Design

**Figure 1:**
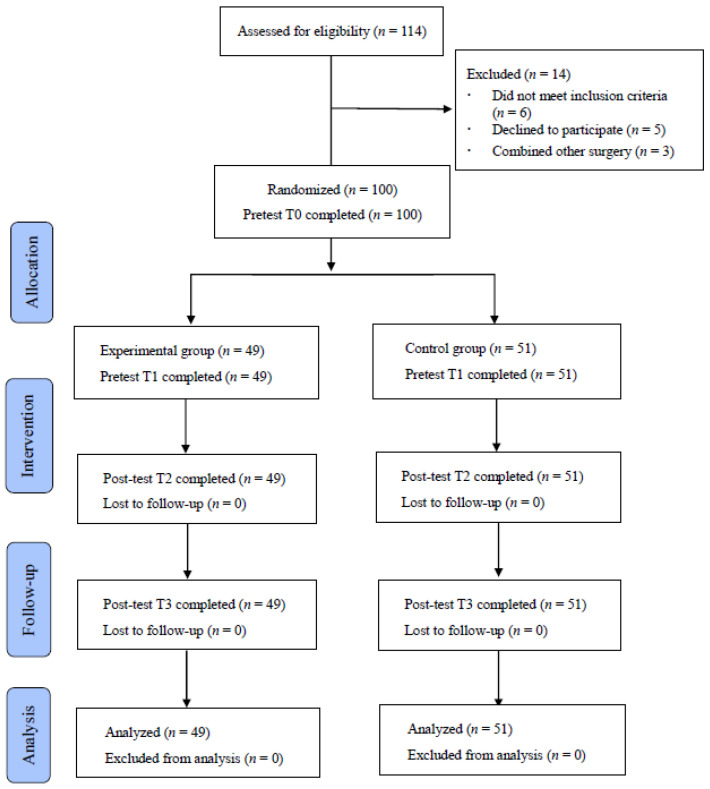
Research flowchart.

A randomized controlled trial with purposive sampling was used for this study. Participants were randomly assigned into experimental and control groups. The assessments were made four times. The first were pre-test T0 on admission day and T1 on day 1 (8 am) after surgery. These two pre-tests were completed before the intervention. The third and fourth were two post-tests, including T2 on day 1 (at 5 pm) and T3 on day 2 (at 5 pm) after the intervention. The study flowchart is shown in Figure 1.

### 2.2. Participants

Participants were enrolled at the cardiothoracic ward of a medical center in central Taiwan between September 2020 and April 2021. Patients were included if they were hospitalized for pulmonary lung disease, or if they underwent thoracoscopic lobectomy, thoracoscopic wedge/partial resection, thoracoscopic segmentectomy, or thoracoscopic sleeve resection. Their physical status of grades I–II was based on the American Society of Anesthesiologists classification system. In addition, the participants’ forearms were without damage or arteriovenous fistula, and the participants were able to verbally communicate in Chinese or Taiwanese. Exclusion criteria included the opioid use of patient-controlled analgesia devices and the diagnosis of malignant pulmonary metastases. Patients with a history of stroke or peripheral neurovascular disorders, with a platelet count lower than 20,000 per microliter, or with cognitive impairment were also excluded. Patients who met the inclusion criteria were referred to the study by thoracic surgeons. A research assistant explained the purpose of the research and the design, patient rights, and ethical issues to the patients. Informed consent was obtained from the enrolled patients who met the inclusion criteria before participation in the study.

### 2.3. Intervention

In addition to routine care, the intervention group received acupressure treatment which involved two acupoints, Neiguan (PC6) [13] and Shenmen (HT7) [14,15]. These acupoints are related to the heart and the pericardium and are commonly used in treating anxiety and insomnia in traditional Chinese medicine. Semen vaccariae uses a skin-colored adhesive tape placed on the two acupoints. Acupressure was performed 20 times (10 s at a time and 2 s released) per acupoint, 3 times each day, for 2 days. The thumb was used to apply pressure vertically on the tape until the participant felt soreness, numbness, or warmth in the area. The adhesive tape was retained in situ for 2 days.

Acupressure was performed by a researcher trained by two experienced experts (a nursing professor and a physician specializing in traditional Chinese medicine) before the initiation of the study. The intervention measures and acupressure procedure were formulated based on clinical reports and expert recommendations [7,16,17,18]. Participants in the control group received regular treatment alone with neither the skin-colored adhesive tape nor any acupressure. The surgeon, primary nurse, and evaluators were blinded to the study groups. All participants received the same pain management (acetaminophen 500 mg, oral, four times a day) throughout their hospitalization period.

### 2.4. Sample Size

The sample size was calculated based on the moderate effect size of 0.25 recommended by Cohen [19]; α error probability was set at 0.05, and power (1–β-error probability) was set at 0.80. A 20% dropout rate was assumed during the study. Accordingly, a sample size of 100 was required for calculations.

### 2.5. Randomization

We randomly assigned participants to two groups using the Random Allocation Software 2.0 (Windows, Seattle, WA, USA) through a random block size of 4. Details of the random allocation were placed in consecutively numbered opaque envelopes. After recruitment, the participants were grouped based on the order of the numbered envelopes.

### 2.6. Measures

The collected data included participants’ demographic characteristics and physical and surgical data and their scores from the Visual Analog Scale (VAS)-A, the State-Trait Anxiety Inventory Y Form (STAI-Y1), and the Shortened General Comfort Questionnaire (SGCQ).

#### 2.6.1. Demographic Information

The participants’ demographic information included sex, age, education, occupation, religious beliefs, marital status, smoking and alcohol drinking status, medical history, and recent 3-year surgical history.

#### 2.6.2. Physical and Surgical Data

Physical and surgical data included 3 items on the physical measurement and 14 items on surgery and postoperative recovery, including the height and weight for the body mass index (BMI), the surgical diagnosis, the surgical site, the surgery duration, the indwelling drains for chest, the time of postoperative return to the patient’s room, the time needed for the patient to get out of bed, the dose of morphine used, the presence of side effects, the use of nonregular pain medication, the days of hospitalization, the hospitalization expense, and the postoperative physical data.

#### 2.6.3. Visual Analog Scale

VAS-A was used in this study as a tool to measure the degree of anxiety. VAS-A is often a straight horizontal line of fixed length (usually 100 mm) with the ends defined as the extreme limits of the measured parameter (anxiety, in our study). The left end (marked as “0”) represents the absence of anxiety, and the right end (marked as “100”) indicates extreme anxiety [20]. Previous studies have shown that VAS-A and STAI are highly correlated in terms of reliability and validity (*r* = 0.55–0.593, *p* < 0.01) as instruments for measuring anxiety [21,22]. VAS-A was accordingly used herein as an anxiety screening tool owing to its validated effectiveness.

#### 2.6.4. State-Trait Anxiety Inventory

The Chinese version of the STAI-Y1 was also used in this study to assess anxiety [23]. The inventory incorporates 20 items rating anxiety on a 4-point Likert scale with “1” indicating “not at all” and “4” suggesting “very much so.” Scores range from 20 to 80. A higher total score correlates with a greater anxiety level. A total score of 20–39 indicates mild anxiety, 40–59 moderate anxiety, and 60–80 severe anxiety [24]. The inventory was previously tested on 306 patients with anxiety disorder. The Cronbach’s α of 0.92 revealed adequate internal consistency, and the 2-week test–retest reliability reached a satisfactory level of 0.76 (*p* < 0.01, 95% confidence interval = 0.376–0.909) [23]. The STAI-Y1 had been used in Taiwanese to measure anxiety levels in many studies [25,26,27].

#### 2.6.5. Shortened General Comfort Questionnaire

The General Comfort Questionnaire, originally developed by Kolcaba and condensed into an abridged version with 28 items, is named SGCQ [28]. This study adopted the Chinese version of SGCQ [29] with each item rated on a 6-point Likert scale ranging from “strongly disagree” to “strongly agree”. Total scores range from 28 to 148 points. With negatively worded items reverse-coded, a higher score indicates a greater degree of comfort. As measured in previous studies, the Cronbach’s α for internal consistency of the scale reached 0.88, and the correlation coefficients of subscales ranged 0.51–0.62. The SGCQ used in the study reported a Cronbach’s α of 0.86.

### 2.7. Data Collection and Ethical Considerations

The study was approved by the institutional review board, and informed consent was obtained before data collection. After participants completed written consent forms, the demographic surveys, VAS-A, STAI-Y1, and SGCQ, were obtained upon admission. The time points of data collection were T0 and T1 for pre-tests and T2 and T3 for post-tests. The VAS-A was measured at T0, T1, T2, and T3. The STAI-Y1 and SGCQ were measured at T0, T1, and T3. The post-test data were measured and recorded immediately after the procedure. Inpatient medical records were collected upon discharge. All documents were anonymized to ensure the participants’ autonomy and confidentiality. The study flowchart is presented in Figure 1.

### 2.8. Data Analysis

The general characteristics of participants were analyzed using descriptive statistics. The homogeneity between the two groups was performed by the independent sample *t*-test and the chi-squared test. The linear mixed model (LMM) for repeated measures was used to examine the outcomes of time–group interactions of VAS-A at four time points, including T0 and T1 for pre-tests and T2 and T3 for post-tests. The outcomes of the time–group interactions of STAI-Y1 and SGCQ at admission day, before intervention, and after intervention were also analyzed. The level of statistical significance was set at 0.05. All data were analyzed using SPSS statistical software (22.0) (IBM Corp, New York, NY, USA).

## 3. Results

### 3.1. Patient Information

During enrollment, 114 patients were hospitalized for thoracoscopic surgery at the host medical center. Of these, 14 were excluded for failing to meet the inclusion criteria. Finally, 100 subjects were enrolled and randomly assigned to the experimental and control groups. None of the enrolled subjects withdrew from the study. The mean age of the included subjects was 60.97 ± 12.19 years, including 61.96 in the experimental and 60.02 in the control group. Their mean BMI was 25.25 (SD = ±6.38) kg/m^2^, including 24.71 (±5.77) in the experimental and 253.77 (±7.01) in the control group. The majority of the included subjects were women (61.0%, 63.3% in the experimental and 58.8% in the control group), and most were married (82%). No difference was found in gender (*t =* 0.207, *p* = 0.649) between the two groups.

In terms of the type of surgery, most of the subjects presented wedge surgery (*n* = 51), followed by lobectomy (*n* = 27) and segmentectomy (*n* = 22). In the experimental group, 15 participants (29.4%) had segmentectomy, 13 had lobectomy (25.5%), and 23 had segmentectomy (45.1%). Subjects included in the experimental and control groups were statistically similar regarding surgical site (*χ^2^* = 3.398, *p* = 0.183). Based on the surgical data, the surgery sites were on the right side of the body in over half of the participants (*n* = 51). Indwelling drainage tubes were pigtail catheters (overall, 72%; experimental subjects, 63.3%; control subjects, 80.4%) and chest tubes (overall, 14%; experimental subjects, 14.3%; control subjects, 13.7%). The mean operative time was 138.57 (±64.45) min, and the mean bleeding volume was 35.86 (±53.73) mL in the experimental subjects. For the control subjects, the mean operative time was 150.71 (±59.99) min, and the mean bleeding volume was 59.69 (±139.03) mL. The independent *t*-test and the chi-squared test revealed no statistically significant differences between the two groups in terms of age, BMI, sex, marital status, recent 3-year surgical history, and surgical data, indicating homogeneity of the baseline demographics and surgery-related data between the subjects in the two groups.

### 3.2. The Effects of Acupressure on Physical Health and Cost-Reducing

Compared with the control subjects, experimental subjects used a lower dose of morphine and developed side effects less frequently (*p* < 0.01). The experimental subjects were able to get out of bed after surgery 163.79 min earlier than the control subjects (*p* < 0.05). Further, the experimental subjects incurred lower health insurance expenses for hospitalization than control subjects; although this difference was not statistically significant, it can be clinically meaningful. As revealed by the results, intervention in the form of acupressure helps to reduce the use of morphine and the incidence of side effects (e.g., nausea and vomiting), and may help shorten the duration and lower the expense of hospitalization. The national health insurance costs of the experimental group are, on average, 10% less (TWD 28,681) than that of the control group (Table 1).

### 3.3. Effects of Acupressure on Mental Health

In terms of the psychological indicators, the average scores of all subjects on the first day after surgery were 21.11 ± 23.36 for VAS-A, 42.53 ± 9.79 for STAI-Y1, and 119.37 ± 18.86 for SGCQ. Subjects in both groups had mild-to-moderate anxiety postoperatively, but the control group showed a higher average STAI-Y1 score than the experimental group (44.67 vs. 40.31). Results of LMM analysis indicated that for VAS-A scores, there was a statistically significant difference in the group-by-time interaction between the two groups (F = 4.218, *p* = 0.008), suggesting an obvious difference in the change in anxiety between the two groups before the intervention. On the first and second days after the intervention (T2 and T3, respectively), a statistically significant decline in regression coefficients was observed in both groups (T2, β = −11.61, *p* = 0.002; T3, β = −18.71, *p* < 0.001). However, the two groups showed no apparent changes in anxiety scores as time progressed.

For STAI-YI scores, there was a statistically significant difference in the pre-test and post-test interactions between the two groups (β = 4.72, *p* = 0.031). Conversely, acupressure did not have a statistically significant difference on comfort (F = 2.953, *p* = 0.057). As indicated by the results of LMM analysis, the average STAI-Y1 score decreased significantly in the experimental group from pre-intervention to T3 (β = −7.33, *p ≤* 0.001). The two groups showed a significant difference in the T3 pre-test and post-test interactions (β = 4.72, *p* = 0.031), indicating an obvious difference in the change in anxiety degree between the groups on the second day post-intervention. However, there was no statistically significant difference in group-by-time interaction between the two groups (F = 2.514, *p* = 0.086), suggesting no obvious difference in the change in STAI-Y1 scores before and after the intervention.

**Figure 2:**
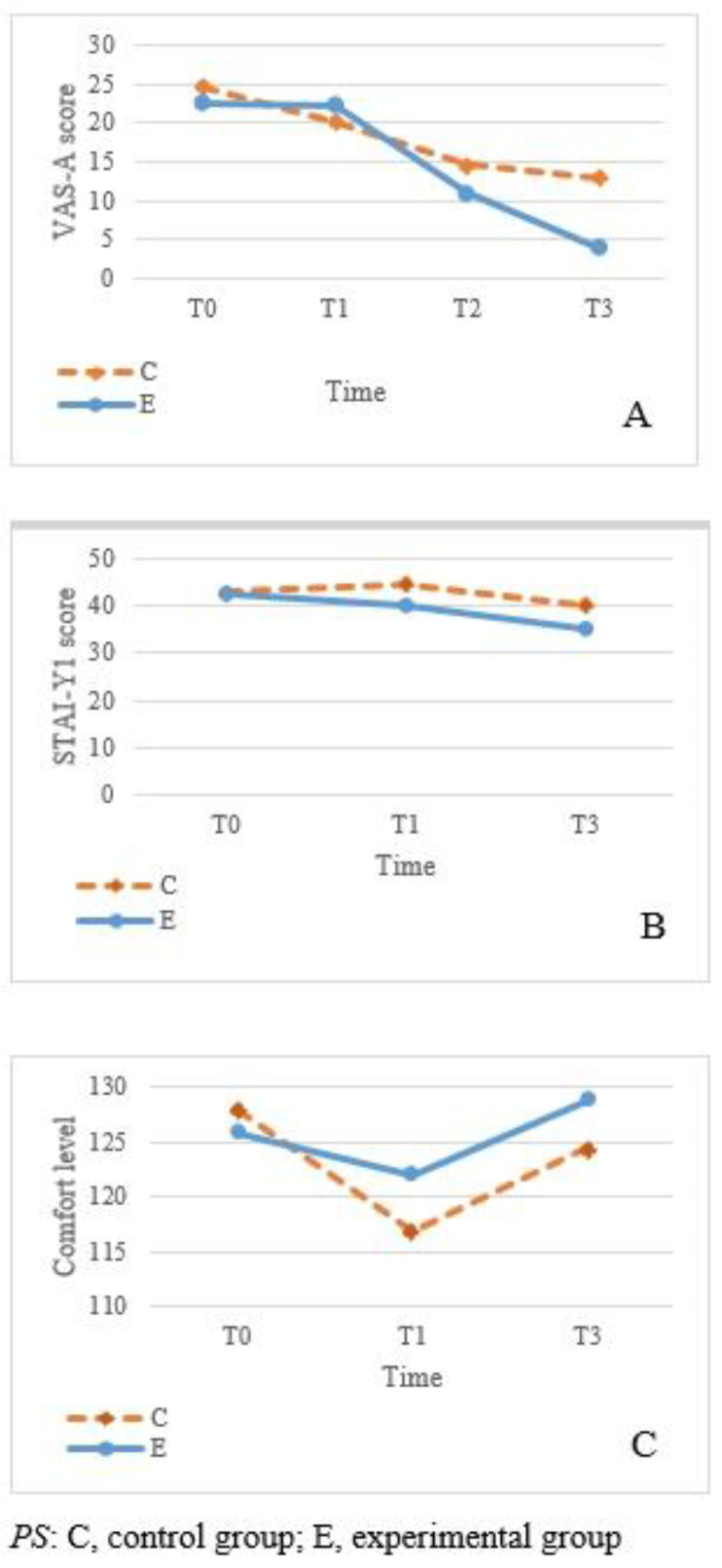
Changes in psychological indicators with time (**A**) VAS-A, (**B**) STA-Y1, and (**C**) comfort.

According to the results of the LMM analysis to assess the effectiveness of acupressure on comfort, the two groups showed no statistically significant difference in terms of group-by-time interaction (F = 2.953, *p* = 0.057), indicating the absence of a significant change in comfort in both groups before and after the intervention. The sense of comfort of the control subjects was at its lowest on T2 after the surgery, but subsequently increased on T3. As time progressed, no significant change in comfort was observed, as shown in Figure 2.

Analysis of the distribution of the VAS-A, STAI-Y1, and SGCQ data of both groups revealed that the decline in the VAS-A and STAI-Y1 scores of the experimental group was apparently greater than that of the control group on the second day after the intervention. Therefore, it can be inferred that the effectiveness of acupressure in reducing the anxiety of patients undergoing thoracoscopic surgery is clinically meaningful. In terms of comfort, the experimental group showed higher scores than the control group before the intervention on T1, and both groups showed an increase in the comfort scores on T3 after the intervention. Nevertheless, the increase in the comfort scores of the experimental group was not higher than that of the control group, suggesting that acupressure is not significantly effective in promoting short-term comfort for patients who have undergone thoracoscopic surgery (Figure 2).

## 4. Discussion

This study investigated the effects of acupressure on the postoperative physical and mental wellbeing of patients undergoing thoracoscopic surgery. The study was the first to use acupressure on patients undergoing thoracoscopic surgery and demonstrated that acupressure can be a supplement to regular treatment and medication for reducing anxiety in patients undergoing thoracoscopic surgery. However, the subjects were found to have moderate anxiety before the surgery, which lasted until the first day after the surgery. Subsequently, anxiety diminished as time progressed, changing from moderate to mild on the second day after the surgery. As anxiety tends to diminish after the end of the surgery, LMM analysis can be used to examine the decrease in the degree of anxiety with time [30]. The anxiety scores of subjects treated with acupressure revealed an obvious decline, and the effectiveness of acupressure in reducing anxiety appeared to increase with time. Therefore, it can be inferred that acupressure exerts a positive influence on reducing the postoperative anxiety of patients undergoing thoracoscopic surgery.

Proper postoperative pain management benefits patients’ early ambulation, reduces analgesia-related side effects, and prevents the development of acute to chronic pain [31,32]. The results of this study indicate that the average time to early ambulation in the experimental group was 163.79 min earlier than that of the control group. The hospital health insurance costs and hospitalization days of the experimental group were lower than those of the control group. Therefore, this study provides evidence that after acupressure intervention, the subjects have less analgesic use, less complication by analgesia, and shorter times for early ambulation, thus promoting pulmonary rehabilitation following thoracic surgery [33], shorter hospital stays, and lower health insurance expenditures.

Acupressure has been reported to alleviate preoperative or pretreatment anxiety [7,12,14,34]. Khoram et al. [12] found that acupressure on the Shenmen and other acupoints was effective in reducing anxiety in patients undergoing heart surgery. Similarly, Valiee et al. [34] reported the positive influence of acupressure in reducing anxiety in patients undergoing abdominal surgery as they found acupressure on the Yingtang and Shenmen acupoints to be effective in stabilizing heart and breathing rates. There are also studies which support the ability of acupressure to reduce anxiety in patients undergoing bone marrow biopsy and aspiration [15]. However, these studies differ from the present study as they focused on preoperative anxiety or anxiety within 30 min after the acupressure intervention.

Acupressure has been found to regulate neurotransmitter concentrations [11] and increase sympathetic nerve activity to stabilize heart rate, thereby helping to alleviate anxiety [13]. Moreover, postoperative experiences may intensify the sense of uncertainty experienced by many patients; acupressure is capable of helping patients develop a coping behavior that can increase their participation in self-care to lower their sense of uncertainty and anxiety [35]. Moreover, based on the results of this study, acupressure can be an effective complementary therapy for reducing persistent anxiety in the two days after thoracoscopic surgery. Although the theory of traditional Chinese medicine confirmed that acupressure can be used to reduce operative-related anxiety, our study found that participation in self-care or increasing companionship with professionals due to the acupressure process may affect this decrease in anxiety. More research can be further explored.

The finding of this study that acupressure results in no significant improvement in the comfort of patients undergoing thoracoscopic surgery is inconsistent with the results of previous studies. A study examining the effects of acupressure on mitigating nausea/vomiting and enhancing comfort reported that practicing acupressure on the Heku acupoint within 12 h after surgery helped improve comfort; however, the study did not examine the correlation between nausea/vomiting and comfort and did not discuss the homogeneity in the time points of measurement between the experimental and control groups [36]. Although no short-term improvement in comfort was observed in this study, subjects who received the acupressure intervention did show a higher comfort level on the second day after the surgery compared with their preoperative status.

In light of this finding, the study revealed that acupressure is clinically meaningful in improving comfort. A qualitative study interviewing patients undergoing surgery in Sweden highlighted the importance of a sense of security for patients during postoperative recovery. Support from family members and friends helps to prevent the feeling of loneliness or isolation in patients, thereby contributing to better outcomes in postoperative recovery [37]. Adequate company and support from family and friends during the immediate postoperative period as a critical factor for improving the overall comfort of patients undergoing surgery may merit further investigation in future studies on the needs of patients undergoing thoracoscopic surgery. The average hospitalization period for thoracoscopic surgery was 5.57 days in this study. Participants were discharged on the fourth day post-operation. Therefore, longer intervention, as well as comfort and quality of life after discharge from the hospital, can be further explored.

## 5. Conclusions

Acupressure is an easy-to-practice, noninvasive complementary treatment that can reduce anxiety in patients undergoing thoracoscopic surgery. Acupressure further helps to reduce the use of morphine and the development of side effects, enables patients to get out of bed earlier, and facilitates recovery to decrease hospitalization duration and health insurance expenses. Therefore, acupressure should be used as an effective complementary intervention for improving the quality of postoperative care.

## Figures and Tables

**Table 1:** Postoperative status of patients in both groups.

Variable	All	Control Group	Experimental Group	*t*/χ^2^	*p*
	*n* = 100	*n* = 51	*n* = 49		
	Mean ± SD	Mean ± SD	Mean ± SD		
Dose of morphine used (mg)	5.75 ± 6.05	7.45 ± 6.81	3.98 ± 4.56	3.01	0.003
Side effect *n* (%)				^a^ 11.92	0.008
NIL	70 (70)	28 (54.9)	42 (85.7)		
Nausea/vomiting	10 (10.0)	7 (13.7)	3 (6.1)		
Dizziness	18 (18.0)	14 (27.5)	4 (8.2)		
Others	2 (2.5)	2 (3.9)	0		
Time needed to get off bed (min)	427.96 ± 388.88	509.69 ± 473.53	342.90 ± 252.68	2.21	0.030
Hospitalization duration (days)	5.57 ± 1.437	5.75 ± 1.383	5.39 ± 1.483	1.25	0.216
Health insurance expense	216,702.66 ± 80,101.95	230,756.33 ± 79,157.77	202,075.37 ± 79,236.15	1.81	0.073
Out-of-pocket expense	62,004.02 ± 46,052.44	64,383.16 ± 53,168.39	59,527.78 ± 37,646.61	0.53	0.601

^a^: Chi-Squared Test; NIL: no side effect.

## Data Availability

These study data are identified participant data. The data that support the findings of this study are available beginning 12 months and ending 36 months following the article publication from the corresponding author, W–FM, upon reasonable request at lhdaisy@mail.cmu.edu.tw.
